# Misdiagnosis of bronchiolitis and misuse of antibiotics – experience from the largest paediatric hospital of Bangladesh

**DOI:** 10.7189/jogh.16.04274

**Published:** 2026-08-14

**Authors:** Farhat Lamisa Kabir, Luthful Kabir ARM, Jahangir Alam, Kazi Selim Anwar, Md Ruhul Amin, Probir Kumar Sarker, Samir Kumar Saha, Rahat Bin Habib, Abid Hossain Mollah, Mohammod Jobayer Chisti

**Affiliations:** 1 Bangladesh Shishu Hospital and Institute (BSHI), Dhaka, Bangladesh; 2 Ad-din Women’s Medical College, Dhaka, Bangladesh; 3 Vision Eye Institute & Hospital, Green Road, Dhaka, Bangladesh; 4 Shahid Syed Nazrul Islam Medical College, Kishoregonj, Bangladesh; 5 Bangladesh Institute of Research and Rehabilitation in Diabetes, Endocrine and Metabolic Disorders; 6 International Centre for Diarrhoeal Disease Research, Bangladesh (icddr,b), Dhaka, Bangladesh

**Keywords:** bronchiolitis, Bangladesh, children, misdiagnosis, misuse of antibiotics, pneumonia

## Abstract

**Background:**

Bronchiolitis remains the commonest acute lower respiratory tract infection in younger children globally. Bronchiolitis is often misdiagnosed as World Health Organization (WHO)-classified pneumonia due to closely related clinical manifestations. Our primary objective was to assess the prevalence of viral bronchiolitis among WHO-classified pneumonia, and the secondary objective was to evaluate their management.

**Methods:**

This was a prospective observational study. Infants (aged <2 years) suffering from WHO-classified pneumonia attending the country’s largest academic paediatric hospital ‘Bangladesh Shishu Hospital and Institute’, Dhaka, during July 2020 through June 2021 were enrolled (n = 60) following systematic sampling (every second child). We revalidated all infants meeting the inclusion criteria (cough, fast breathing and/or chest in-drawing) for bronchiolitis assessment using clinical, radiological, haematological, biochemical, and microbiological analyses. Nasopharyngeal aspirate (NPA) was subjected to polymerase chain reaction (PCR) assay for respiratory syncytial virus (RSV), influenza A, and influenza B viruses. Clinical features, treatment modalities, mode of discharge, and length of hospital stay (LOS) were subjected to differentiate bronchiolitis from WHO-classified pneumonia.

**Results:**

PCR of NPA of 60 WHO-classified pneumonia cases yielded RSV in 41 (68%) who had pertinent clinical symptoms and signs suggestive of bronchiolitis, attested by benign chest radiography (85%). Further, their mean (x̄) and standard deviation (SD) complete blood count (x̄ = 15,488; SD = 1874/cm) with x̄ = 36% (SD = 11) neutrophil) and C-reactive protein (x̄ = 4.49 (5.88) mg/L cut off reference value >5 mg/L) were indicative more as non-bacterial infections favouring RSV bronchiolitis. In contrast, all 19 non-RSV cases did not differ in clinical findings, as attested by haematological, microbiological and radiographical features, suggesting bronchiolitis-like illness, further supported by disease outcome, discharge pattern and LOS (x̄ = 4.7; SD = 2.29) days). Nevertheless, antibiotics were given in 95% of all 60 cases.

**Conclusions:**

There was a high proportion of bronchiolitis among WHO-classified pneumonia, and antimicrobials were prescribed to treat those cases. The findings need the attention of policymakers to address the overlap between WHO-classified pneumonia and bronchiolitis and minimise the potential overuse of antibiotics.

Acute lower respiratory tract infection (ALRTI) [1], comprising of both bronchiolitis and pneumonia among children aged <2 years [1], reportedly remains a major public health issue [2]. Bronchiolitis **– **a serious respiratory illness commonly affecting infants/young children [1], claiming 30% of global paediatric admissions, usually in low- and middle-income countries (LMICs), especially in the Afro-Asian region [3,4].

Clinically, both bronchiolitis and pneumonia apparently remain similar, though they differ in diagnosis and treatment. Bronchiolitis, a viral disease, has a succinct clinical case definition [5]. Bronchiolitis is a disease of respiratory distress associated with cough, wheeze or crackles on auscultation in infants aged <2 years, attested by radiological hyperinflation and/or increased translucency, but no evidence of any consolidation [6]. Unlike World Health Organization (WHO) classified pneumonia, which requires antibiotics, bronchiolitis is managed with supportive care only [6–8].

There is a great impact of implementing the Integrated Management of Childhood Illness (IMCI) programme in reducing childhood pneumonia-related mortality globally and regionally [9]. The mortality from childhood pneumonia has reduced significantly from 150,000 to 24,000 from 1990 to 2023 in Bangladesh [10]. However, the number of deaths from childhood pneumonia is still high and has scope for further reduction [2,11].

While bronchiolitis and pneumonia are easily confused, IMCI guidelines provide no specific instructions for differentiating between them. Consequently, clinicians working in health facilities in LMICs, including Bangladesh, do not differentiate bronchiolitis from WHO-classified pneumonia. Moreover, the IMCI programme has been designed for field-level health workers, as well as for nurses and paramedical staff in first-level peripheral health facilities. But this has been extrapolated by physicians working even in tertiary-care hospitals. This potentially results in overdiagnosis of pneumonia and indiscriminate use of antibiotics [12]. Although the WHO, in its pneumonia guideline, provided a brief but precise description for differentiating bronchiolitis from pneumonia for hospital physicians, this is not duly followed by clinicians [5,7,12] in most hospitals in LMICs, including Bangladesh.

In an earlier study, 65% of 100 cases of WHO-classified severe pneumonia tested positive for viruses that cause bronchiolitis [13]. Thus, the primary objective of this study was to evaluate the prevalence of viral bronchiolitis among WHO-classified pneumonia. Our secondary objective was to assess their management.

## METHODS

Infants suffering from WHO-classified pneumonia, admitted to the study site, were enrolled following systematic sampling (every second child). The study was conducted in the Department of Pediatric Respiratory Medicine, Bangladesh Shishu (paediatrics) Hospital and Institute (BSHI), Dhaka, Bangladesh. BSHI is the oldest (established in 1983) and largest (700-bed) children's hospital, a government-supported, tertiary-level public hospital for children. All the laboratory investigations were conducted in the Child Health Research Foundation laboratory (funded by the Gates Foundation). One year (July 2020 to June 2021), to cover all seasons in Bangladesh.

We enrolled children aged 2–24 months of either sex who were hospitalised with cough, fast breathing and/or chest indrawing and had written informed consent. Children who were excluded from the study had congenital heart disease (CHD), were critically sick (required cardio-pulmonary resuscitation), were receiving prior antibiotics, had suspected pulmonary tuberculosis, foreign body aspiration, cystic fibrosis, primary immunodeficiency, were COVID-19 positive, and did not give consent.

**Figure 1:**
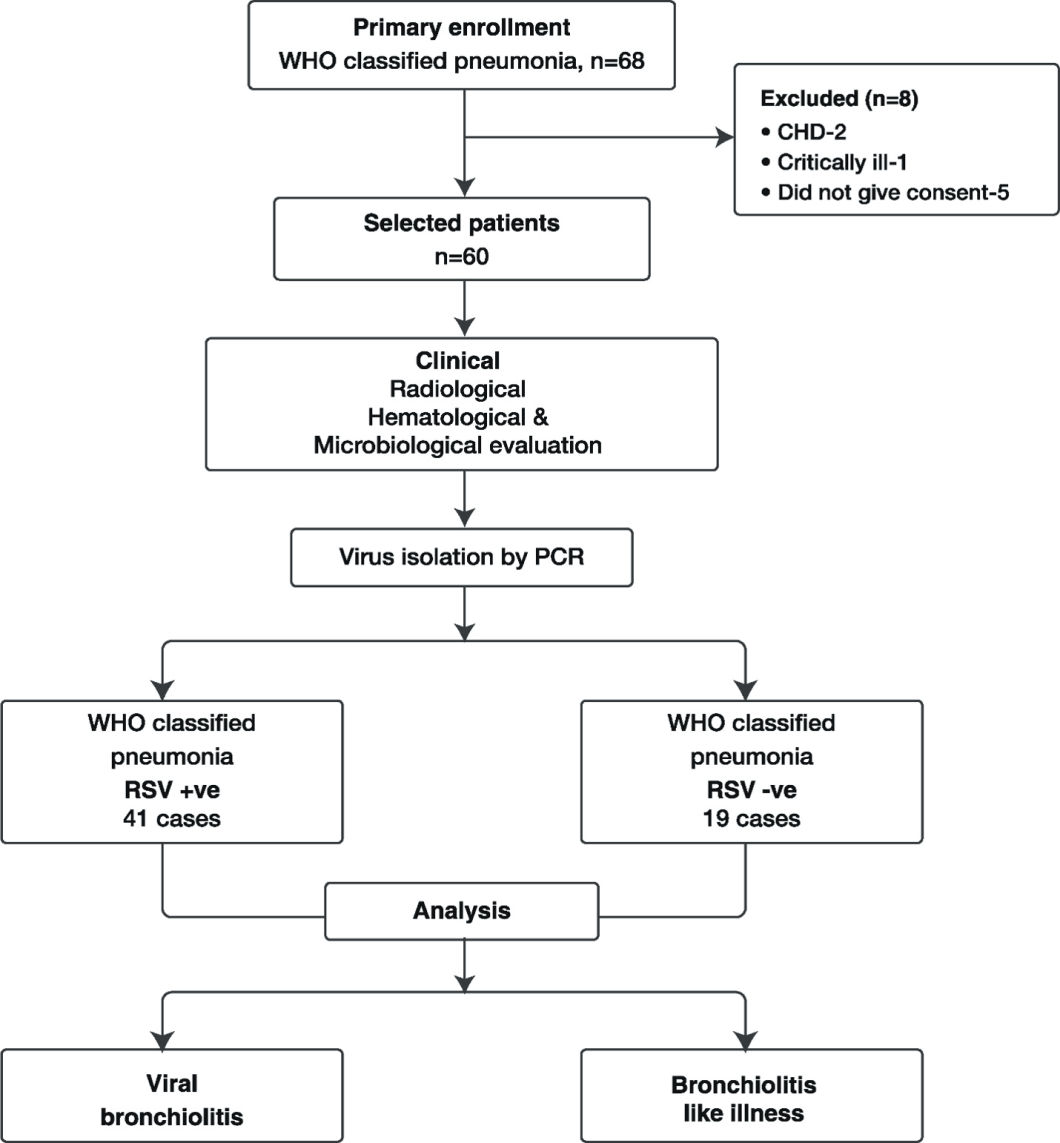
Flowchart of the study.

We screened every second child with WHO-classified pneumonia features (history of cough with age-specific fast breathing, and/or lower chest wall indrawing). After screening, we evaluated all screened children for eligibility. Those who fulfilled the eligibility criteria were enrolled in the study during the study period (**Figure 1**). Face-to-face interviews with mothers/caregivers of enrolled children were conducted using a pretested structured questionnaire. A detailed history was taken, and a thorough physical examination was performed. The outcome measures were the prevalence of bronchiolitis among WHO-classified pneumonia cases, treatment received, length of stay (LOS), and discharge pattern from the hospital.

Each child was subjected to complete blood count with peripheral blood film, C-reactive protein (CRP) and blood culture and sensitivity (C/S) for *Streptococcus pneumoniae*, *Haemophilus* *Influenzae*, *Staphylococcus aureus* in Becton, Dickinson bacterial detection fluorescence extreme with peds plus culture vials before giving antibiotics, chest x-ray antero-posterior view and lateral (if needed) to be reported by a radiologist and paediatric pulmonologist; nasopharyngeal aspirate (NPA) for isolation of respiratory syncytial virus (RSV), Influenza A and B virus by PCR method. Instruments used to collect data were a pre-structured questionnaire, a wristwatch, a stethoscope, and a pulse oximeter.

The patients’ management in the ward was meticulously documented. The patients were admitted under different consultants and treated according to the directions given by the respective consultants. There was no uniform management protocol followed for the admitted patients. The modes of treatment were oxygen therapy (when peripheral capillary oxygen saturation <92%), antibiotics, nebulisation with salbutamol, 3% hypertonic saline, ambroxol hydrochloride, and nasal clearing. The children were followed for the following outcomes: LOS, discharge with advice, discharge on request, leave against medical advice (LAMA), absconded, referral to paediatric intensive care unit, and expired.

### Operational definitions

Bronchiolitis is defined as the ‘WHO-classified pneumonia features’ with wheeze/rhonchi and benign chest radiography plus RSV positivity. Bronchiolitis-like illness is defined as the ‘WHO-classified pneumonia features’ with wheeze/rhonchi and benign chest radiography plus RSV negativity. Benign chest radiography comprises hyperinflation, peribronchial cuffing, increased interstitial markings, ground-glass opacity and increased translucency. Pathological chest radiography showed lobar consolidations, patchy opacities, and a confluence of opacities, consistent with airspace disease and/or adjacent airway disease.

### Ethical considerations

Prior to the commencement of the study, the research protocol was approved by the Ethical Review Committee of Bangladesh Institute of Child Health, Dhaka (approval number BICH-ERC-06-01-2020). The aims and objectives, risks, and benefits of this study were explained to the children's parents in an easily understood local language using a consent form. The consent form assured that all information and records would be kept confidential. They were at liberty to withdraw from the study at any time if they wished, and this did not affect patient management. Once the legal guardians of each child understood and agreed to the contents of the consent form, informed written consent was obtained from them.

All patient information was kept confidential under the principal investigator's responsibility. Only the investigator and the ethical review committee had access to the collected data. The identities of the patients were not disclosed during the analysis or publication of the results of this study.

### Statistical analysis

All rechecked data were entered into an International Business Machines computer and processed for statistical analysis using SPSS version 23.0 for Windows (SPSS Inc., Chicago, Illinois, USA).

First, using descriptive statistics measures of dispersion (range, variance, standard deviation, mean/quartile deviation), the data distribution was understood to get average values. Frequency distributions were primarily reported, expressed as numbers and percentages for categorical variables (gender, clinical features) and as means for quantitative variables. Inferential statistics were then used to determine *P*-values by unpaired *t* test (for continuous variables with homogeneous data), Mann-Whitney test (for continuous variables with non-homogeneous data), χ^2^ test for categorical variables, and Fisher exact test for 2 × 3 tables. For all statistical tests, a *P*-value <0.05 was considered statistically significant.

## RESULTS

**Figure 2:**
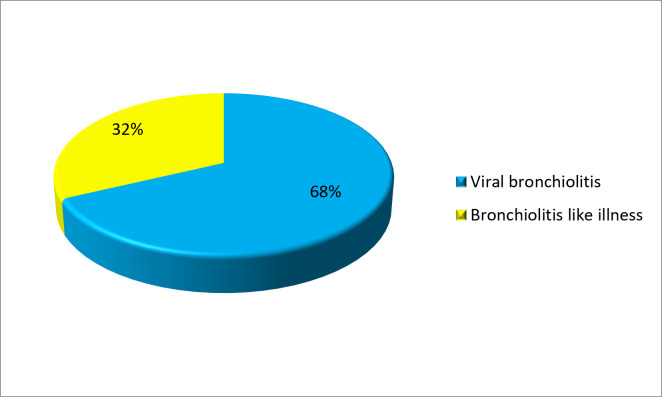
World Health Organization classified pneumonia perceived to be viral bronchiolitis and bronchiolitis-like illness (n = 60).

Among 60 WHO-classified pneumonic children, 41 (68%) were RSV-positive, and 19 (32%) were RSV-negative (**Figure 2**). The age of all WHO-classified pneumonia children was x̄ = 5.7 (3.7) months; there were 91.7% infants up to 12 months and only 8.3% between 13–24 months. Further, 66% were male, and 33% were female. A total of 55 (92%) babies were born term and 54 (90%) had appropriate birth weight. However, no significant differences were observed across the socio-demographic variables (**Table 1**).

**Table 1:** Demographic features of World Health Organization-classified pneumonia*

Demographic features	WHO-classified pneumonia (n = 60)			*P*-value
	**Total**	**Based on RSV positivity**		
		**RSV positive (n = 41)**	**RSV negative (n = 19)**	
Age in years, x̄ (SD)	5.77 (3.71)	5.29 (3.01)	6.70 (4.90)	0.18
Proportionally distributed age groups in months				0.31
*2–12*	55 (92.00)	39 (65.00)	16 (27.00)	
*13–24*	5 (8.00)	2 (3.00)	3 (5.00)	
Body weight in kg, x̄ (SD)	6.5 (1.98)	6.12 (1.54)	7.31 (2.56)	0.07
Gender				0.77
*Male*	40 (66.70)	28 (68.30)	12 (63.20)	
*Female*	20 (33.30)	13 (31.70)	7 (36.80)	
Birth (gestational outcome)				1.00
*Term*	55 (92.00)	37 (90.20)	18 (94.70)	
*Preterm*	5 (8.00)	4 (9.80)	1 (5.30)	
Birthweight				0.46
*Appropriate*	54 (90.0)	37 (90.20)	17 (89.50)	
*Low*	6 (10.0)	4 (9.70)	2 (10.50)	

RSV – respiratory syncytial virus, SD – standard deviation, WHO – World Health Organization, x̄ – mean

*Presented as n (%) unless specified otherwise.

All the children presented with cough, difficulty breathing, fast breathing, restlessness, inconsolable crying, feeding difficulty, sleeping difficulty, and lapse of social smile; a stuffy/blocked nose was present in 71.6% of cases, and a blocked nose in 53% of cases. (**Table 2**).

**Table 2:** Clinical features (symptoms) of World Health Organization-classified pneumonia*

Symptoms	WHO-classified pneumonia (n = 60)		*P*-value
	**RSV positive**	**RSV negative**	
Fever (n = 03)	0.00	01 (2.43)	0.026
Child age in months respiratory rate/min, x̄ (SD)			
*2–12*	58.75 (4.20)	58.97 (3.82)	0.518
*13–24*	46.00 (2.80)	46.00 (2.80)	0.329
Cough (n = 60)	41 (100.00)	19 (100.00)	1.00
Difficult breathing (n = 60)	41 (100.00)	19 (100.00)	1.00
Fast breathing (n = 60)	41 (100.00)	19 (100.00)	1.00
Restlessness (n = 60)	41 (100.00)	19 (100.00)	1.00
Inconsolable cry (n = 60)	41 (100.00)	19 (100.00)	1.00
Lapse of social smile (n = 60)	41 (100.00)	19 (100.00)	1.00
Feeding difficulty (n = 60)	41 (100)	19 (100.00)	1.00
Sleeping difficulty (n = 60)	41 (100.00)	19 (100.00)	1.00
Stuffy/blocked nose (n = 43)	31 (75.60)	12 (24.40)	0.37
Runny nose (n = 32)	22 (53.70)	10 (46.30)	1.00

RSV – respiratory syncytial virus, SD – standard deviation, WHO – World Health Organization, x̄ – mean

*Presented as n (%) unless specified otherwise.

The mean temperature was 98.4°F; the mean respiratory rate (RR) of the 2–12 months’ age group was 59 per minute, and that of beyond 12 months was 48 per minute. There was lower chest wall in-drawing in 95% of cases, wheeze in 100% of cases, and crepitations in 20% of cases. The mean oxygen saturation was 90 %. The liver was palpable in 73% of cases. (**Table 3**).

**Table 3:** Clinical features (signs) of World Health Organization-classified pneumonia*

Signs	WHO-classified pneumonia (n = 60)	WHO-classified pneumonia, RSV positive (n = 41)	WHO-classified pneumonia, RSV negative (n = 19)	*P*-value
Temperature in °F, x̄ (SD)	98.38 (1.13)	98 (0.62)	99 (1.80)	0.06
Child age in months respiratory rate/min, x̄ (SD)				
*2–12*	58.76 (4.20)	58.97 (3.82)	58.25 (5.20)	0.52
*13–24*	48.00 (2.80)	46.00 (2.80)	49.33 (2.30)	0.33
Heart rate per minute, x̄ (SD)	128 (15)	128 (14)	126 (17)	0.43
Nasal flaring	29 (48.30)	18 (43.90)	11 (57.90)	1.00
Chest in-drawing	57 (95)	39 (95.10)	18 (94.70)	0.69
Wheeze/rhonchi	60 (100.00)	41 (100.00)	19 (100.00)	0.23
Crackles	12 (20%)	08 (19.50%)	04 (21.10%)	1.00
SpO_2_, x̄ (SD)	89.82 (5.54)	90.70	87.80	0.39
Palpable liver	44 (73.30)	30 (73.10)	14 (73.70)	0.93

RSV – respiratory syncytial virus, SD – standard deviation, SpO_2_ – peripheral capillary oxygen saturation, WHO – World Health Organization, x̄ – mean

*Presented as n (%) unless specified otherwise.

Radiological evaluation showed hyperinflation (52%), peribranchial cuffing (50%), increased translucency (42%), ground-glass opacity (GGO) (23%), and increased interstitial markings (22%). ‘Benign chest radiographic’ findings were found in 85% of cases in various combinations, and ‘pathologic radiographic’ findings (lobar consolidation, patchy opacities, and confluence of opacities) were observed in 15% of cases (Table S1 in the **Online Supplementary Document**). Median and interquartile values of haemoglobin, white blood cell (WBC) count, neutrophil, and CRP between RSV-positive and RSV-negative WHO-classified pneumonia cases without observing any statistical difference (Table S2 in the **Online Supplementary Document**).

Among nine ‘pathologic chest radiographic’ cases, six were found to be RSV positive, denoting that they were in fact bronchiolitis or viral pneumonia (Table S3 in the **Online Supplementary Document**). The WHO-classified pneumonia cases were managed with oxygen therapy in 46 (77%) cases and antibiotics in 95% of cases, with 53% receiving ceftriaxone.

Nebulised therapy was given in 34 (57%) cases. Nebulised salbutamol, ipratropium bromide, and normal saline were given in 21 (35%), and nebulised ipratropium bromide and normal saline in 13 (22%). Hydrocortisone was administered in 13 (22%) cases. The mean length of hospital stay was x̄ = 4.7 (SD = 2.29) days in all cases, x̄ = 4.68 (SD = 2.23) days in RSV-positive cases, and x̄ = 4.89 (SD = 2.47) days in RSV-negative cases, with antibiotics given in 57 (95%) cases. Importantly, 40 (98%) of RSV-positive cases received antibiotics without any justification. A total of 52 (87%) cases with WHO-classified pneumonia were discharged with improvement, and eight (13%) were discharged on request. There were no referrals, LAMAs, or deaths among the study population.

## DISCUSSION

This small study highlights the current conditions of likely antibiotic overuse in the largest children's hospital in Bangladesh. Among WHO-classified pneumonia cases, more than two-thirds were RSV-positive, with clinical features of bronchiolitis, benign chest radiography, normal WBC count, and normal CRP, and no growth in blood culture.

Likewise, all 19 non-RSV cases did not differ in clinical findings, haematological indices, microbiological culture and radiographic features, potentially simulating bronchiolitis-like illness. These were further supported by disease outcome, discharge pattern and mean length of hospital stay. 

In 85% of cases, there were benign radiological features. Moreover, use of antibiotics in our study population was found in 95% of cases.

Why were the aforementioned cases bronchiolitis? Bronchiolitis is a clinical diagnosis and requires a clinician to recognise the signs and symptoms of viral lower respiratory tract infection in young children [14]. We adopted diagnostic criteria for a coryzal prodrome, cough followed by breathing difficulty, fast breathing, lower chest wall indrawing, and wheeze, which we use in Bangladesh [6,15]. The mean age of our cases was 5.77 (SD = 3.71) months, consistent with the peak incidence of bronchiolitis between three and six months of age [16]. All our cases had cough (100%), history of stuffy/runny nose (53%), blocked nose (72%), breathing difficulty (100%), fast breathing (100%), and wheeze (100%), all of which are suggestive of bronchiolitis. Other features encompassed restlessness (100%), inconsolable cry (100%), lapse of social smile (100%), including feeding (100%) and sleeping difficulty (100%). Signs of bronchiolitis were also consistent with published data on fast breathing (100%), chest indrawing (95%), wheeze (100%), crackles (20%), and hypoxemia [6]. The mean peripheral capillary oxygen saturation of cases was less than 90% (x̄ = 89.82 (SD = 5.54) as found in another study [6]. All of our study cases were afebrile (mean temperature 98.4°F). However, the literature suggests that one-third of infants with bronchiolitis experience fever with a temperature <39°C in the early stage of illness [16].

We thus also categorised the radiological features of our bronchiolitis cases as ‘benign chest radiography’, comprising hyperinflation, peribronchial cuffing, increased interstitial markings, ground-glass opacity, and increased translucency. ‘Pathological chest radiography’ consisted of lobar consolidation, patchy opacities and confluence of opacities denoting airway disease and/or adjacent airspace disease [17]. The chest radiograph showed multiple combinations of hyperinflation, peribronchial cuffing, increased interstitial markings, ground-glass opacity, and increased translucency (hyperaeration). Considering the benign/simple chest radiologic findings, our cases are consistent with bronchiolitis [6,18].

Further consideration of the hemogram test, CRP, and bacteriology, along with the clinical features, indicates that all cases who were RSV-positive and/or negative corroborate the diagnosis of viral bronchiolitis-like illness, respectively. Fourteen cases showed GGO, with a mean WBC count of 22,673/cmm, a mean CRP of 2.8 mg/L, and negative blood cultures, suggesting bronchiolitis, potentially not associated with bacterial co-infection [19].

Bacterial pneumonia in children is associated with moderate-to-high-grade fever, fast breathing, lower chest wall indrawing, features of consolidation, crackles, a polymorphonuclear response in the blood, increased levels of acute-phase reactants such as CRP and procalcitonin, and alveolar consolidation on chest radiograph [20]. Most of our cases were afebrile; the mean temperature was 98.38°F. A plain chest radiograph is a part of the investigation in children aged <5 years for the diagnosis of pneumonia [20]. Laboratory features are evidenced by leucocytosis (>15,000/cmm), elevated CRP (>5.0 mg/L), and infrequently positive blood cultures for *Streptococcus pneumoniae*, *Haemophilus influenzae*, and *Staphylococcus aureus.* Pathological radiological findings, either patchy opacity (four cases), confluence of opacity (four cases), and lobar consolidation (one case) were observed in nine cases. Out of four cases of patchy opacity, the mean WBC count was 16,000/cmm, mean CRP 11.42 mg/L and RSV was positive in three cases. A confluence of opacities was observed in four cases, with a mean WBC of 13,350/cmm, a mean CRP of 3.32 mg/L, and two RSV-positive cases. One case of lobar consolidation had a WBC count of 10,900/cmm, CRP 3.00 mg/L and was RSV positive. Lobar and segmental consolidation can be observed in RSV/ viral pneumonia [21]. All the cases were negative for bacterial growth. Of the nine cases with pathological radiology, six were RSV-positive, indicating viral bronchiolitis or pneumonia.

Viral pneumonia is more commonly associated with young age, breathlessness and wheezing [22]. RSV is a very frequent viral respiratory pathogen of the young (<5 years), with a significant portion of young toddlers having been infected before two years of age. The radiographic feature is characterised by hyperinflation, bronchial wall thickening and focal areas of atelectasis [6]. However, the chest radiograph may show abnormal findings, including bilateral patchy areas of consolidation, interstitial lung disease, diffuse airspace consolidation, and lobar consolidation in viral pneumonia. The abnormalities were predominantly bilateral and more frequent in the lower zones than in other regions [21]. Though our four cases with patchy opacities were characterised by leucocytosis (x̄ = 16,000/cmm) and raised CRP (x̄ = 11.42 mg/L), three were RSV-positive, and all were negative for bacterial blood culture, suggesting bronchiolitis or viral pneumonia. However, secondary bacterial infection may also be a possibility. The other three cases, though RSV-negative, had no leucocytosis, low CRP and were negative for bacteria, potentially suggesting viral pneumonia; however, the chance of concurrent bacterial pneumonia cannot be excluded.

Of 60 WHO-classified pneumonia cases we studied, 41 (68%) were RSV bronchiolitis based on clinical, biochemical, radiological, and hemogram findings. NPA isolation yielded only RSV on PCR. Our previous experience showed a high prevalence (65%) of viral infection in WHO-classified severe pneumonia, where the virus was RSV (63%), influenza A (5%) and adenovirus in one case and mixed viral infection in two cases, out of a total of 100 WHO-classified severe pneumonia cases. The viruses were detected using a rapid immunochromatographic kit [13]. A total of 59% of bronchiolitis cases were diagnosed, of which 45 were any-virus-positive (viral bronchiolitis). In another study, a very large sample of 632 cases was used [23]. This signified a high prevalence of bronchiolitis, 69% (n/N = 438/632), among lower respiratory tract infections, based on WHO criteria and symptom onset ≤4 days prior to hospitalisation. The diagnosis was made on clinical symptoms, routine laboratory tests and chest radiography and the physicians were unaware of viral diagnostic tests. There were an additional 26% of cases of pneumonia and 3% with combined bronchiolitis and pneumonia. We observed that physicians underdiagnosed bronchiolitis and overdiagnosed pneumonia in our community in Bangladesh. In a total of 3484 under-five children, bronchiolitis was diagnosed in 4.3% of cases, down from 21.4%, and pneumonia in 17% of cases, increased from 11.5% [24]. These studies potentially accounted for a large proportion of WHO-classified pneumonia cases incorporating bronchiolitis. Thus, physicians are often hesitant to apply his/her clinical skills to differentiate bronchiolitis from pneumonia.

Clinical cases were selected strictly in accordance with the WHO classification of childhood pneumonia. The mothers of the cases were interviewed, a thorough physical examination was performed, and the cases were prepared for investigation by the principal/co-investigators (paediatricians). Each and all eligible cases underwent all essential investigations (CBC, CRP, chest x-ray, blood cultures for *Streptococcus pneumoniae*, *Haemophilus influenzae*, and *Staphylococcus aureus*, and NPA for RSV and influenza virus types A and B). All laboratory investigations were performed at the Child Health Research Centre, one of the country’s high-standard laboratories serving BSHI.

The small sample size is our main limitation, resulting in underpowered analyses, especially in several subgroup comparisons. We did not perform NPA tests for other viruses causing bronchiolitis, such as rhinovirus (common cold), human metapneumovirus, adenovirus, or human bocavirus, nor did we perform blood culture for bacteria, other than the three most common causes of childhood pneumonia (*Streptococcus pneumoniae*, *Haemophilus influenzae*, and *Staphylococcus aureus*).

Physicians are often reluctant to diagnose and distinguish bronchiolitis from pneumonia, as the WHO criteria for bronchiolitis are not well understood. This observation suggests that policymakers in developing countries may help clinicians improve their skills in correctly identifying and treating various respiratory disorders.

There is growing consensus that physical examination skills have been greatly deteriorating over the years. Importantly, the introduction of WHO criteria for the diagnosis and management of pneumonia in the IMCI guideline contributed significantly to reducing under-five mortality. On the other hand, the lack of availability of distinguishing features of bronchiolitis from WHO-classified pneumonia in the IMCI guideline commonly leads to under-diagnosis of bronchiolitis and often persuades clinicians to likely overuse antibiotics, potentially leading to bacterial resistance, increasing the burden of chronic disease, raising the cost of health services and the development of side effects.

## CONCLUSIONS

Our data suggest that, despite the high prevalence of RSV bronchiolitis among WHO-classified pneumonia cases, the likely overuse of antibiotics was high. The findings underscore the imperative for policymakers to resolve this enduring debate over the differentiation of bronchiolitis from WHO-classified pneumonia, which may help minimise the potentially unnecessary use of antimicrobials, especially in infants.

## Additional material


Online Supplementary Document

## Data Availability

**Data availability:** The data sets we used and analysed are available from the first author or corresponding author upon reasonable request, in line with institutional and ethical requirements.
