# Supplementary material for: Misdiagnosis of bronchiolitis and misuse of antibiotics – experience from the largest paediatric hospital of Bangladesh
Source: J Glob Health. 2026 Aug 14;16:04274. doi: 10.7189/jogh.16.04274 (PMC13473931; doi:10.7189/jogh.16.04274)
Supplement: Supplementary file 1 [file jogh-16-04274-s001.pdf]

Supplement to: Kabir FL, Kabir ARML, Alam J, Anwar KS, Amin MR, Sarker PK, Saha SK, Habib RB, Mollah AH, Chisti MJ. Misdiagnosis of bronchiolitis and misuse of antibiotics – experience from the largest paediatric hospital of Bangladesh. J Glob Health 2026;16:04274.

Table S1 Radiological features of WHO classified pneumonia

Radiological features	WHO pneumonia n=60 (100%)	WHO classified pneumonia RSV positive n=41 (68.3%)	WHO classified pneumonia RSV negative n=19 (31.7%)	p value
Benign chest radiography (51, 85%)				
Hyperinflation	31 (51.6%)	20 (48.78%)	11 (63.20 %)	.511
Peribronchial cuffing	30 (50%)	24 (58.50 %)	6 (31.60%)	
Increased interstitial markings	13 (21.6%)	09 (21.9%)	04 (21.05 %)	1.00
Ground glass opacity	14 (23%%)	11 (26.82%)	03 (15.78 %)	.75
Increased translucency	25 (41.6 %)	13 (31.70%)	14 (73.68%)	.083
Pathologic chest radiography (9, 15%)				
Patchy opacities	4 (6.70 %)	3 (7.3 %)	1 (5.2%)	01
Confluence of opacity	4 (6.70%)	2 (10.5%)	2 (10.5 %)	0.71
Consolidation	01 (1.0%)	01 (2.4%)	00	1.00**

* p value reached from chi-square test, **p value reached from fisher exact test

Table S2: Laboratory features of WHO classified pneumonia

Hemogram	*WHO* classified *pneumonia n=60 (100%)*	*WHO* classified *pneumonia RSV +ve 41 (68%)*	*WHO* classified *pneumonia RSV -ve 19(32%)*	*p value*
Hb (g/dl)	10.55 (9.6-11.4)	10.40 (9.3-11.35)	10.90 (10.1-11.6)	0.441

WBC /cmm (median, IQR)	10,900 (9125-15850)	10900 (8900-14250)	12000 (9200-17400)	0.154
Neutrophil (%)	34 (25-45)	35 (29-45)	34 (20-37)	0.0.504
Lymphocyte (%)	60 (50-69)	60 (50-66)	61 (50-72)	0.907
Platelet count /cmm (median, IQR)	414500 (336250-524750)	406000 (320500-514500)	423000 (336000-541000)	0.656
Normocytic normochromic	58.3	58.5	57.9	0.988
CRP mg/L#	1.9 (0.82-5.1)	2.0 (0.85-5.5)	1.5 (0.70-5.1)	0.375

* p value reached from student 't' test

Normal cut off point of CRP ≤5.0 mg/L and for leukocytosis ≤15000/cmm

Table-S3: Laboratory findings of different radiological features among WHO classified pneumonia

Benign Chest Radiographic findings (n=51)				
Radiological findings	Mean WBC (cmm)	Mean CRP (mg/L)	No. of RSV + cases	No. of RSV - cases
Hyperinflation	11654	4.13	20	11
Peribronchial cuffing	12667	4.18	24	6
Increased translucency	11587	3.98	14	11
Ground glass opacity	22673	2.8	11	3
Increased interstitial marking	11092	2.45	9	4
Pathologic chest radiographic findings (n=9)				
Radiological findings	Mean WBC (cmm)	Mean CRP (mg/L)	No. of RSV +ve cases	No. of RSV -ve cases
Patchy opacity	16000	11.42	3 (7.3 %)	1 (5.3 %)
Confluence of opacity	13350	3.32	2 (4.8)	2 (10.6 %)
Lobar consolidation	10900	3.0	01	00
